# Capuchin monkeys do not show human-like pricing effects

**DOI:** 10.3389/fpsyg.2014.01330

**Published:** 2014-12-02

**Authors:** Rhia Catapano, Nicholas Buttrick, Jane Widness, Robin Goldstein, Laurie R. Santos

**Affiliations:** ^1^Comparative Cognition Laboratory, Psychology Department, Yale UniversityNew Haven, CT, USA; ^2^Department of Agricultural and Resource Economics, University of California at DavisDavis, CA, USA

**Keywords:** pricing effects, capuchin monkeys, *Cebus apella*, comparative cognition, decision bias, evolutionary origins

## Abstract

Recent work in judgment and decision-making has shown that a good's price can have irrational effects on people's preferences. People tend to prefer goods that cost more money and assume that such expensive goods will be more effective, even in cases where the price of the good is itself arbitrary. Although much work has documented the existence of these pricing effects, unfortunately little work has addressed where these price effects come from in the first place. Here we use a comparative approach to distinguish between different accounts of this bias and to explore the origins of these effects. Specifically, we test whether brown capuchin monkeys (*Cebus apella*) are also susceptible to pricing effects within the context of an experimentally trained token economy. Using a capuchin population previously trained in a token market, we explored whether monkeys used price as an indicator of value across four experiments. Although monkeys demonstrated an understanding of which goods had which prices (consistently shifting preferences to cheaper goods when prices were increased), we observed no evidence that such price information affected their valuation of different kinds of goods. These results suggest that human pricing effects may involve more sophisticated human-unique cognitive capacities, such as an understanding of market forces and signaling.

## Introduction

Congratulations! You have just won a bottle of wine. You have two options: a 2001 pinot noir that costs $10 or another pinot noir from the same year that costs $50. Which do you choose? You probably chose the more expensive wine. Indeed, when given a choice like this, people tend to pick the most expensive options, whether those options involve alcohol (e.g., Jacoby et al., 1971; Plassmann et al., 2008), meat (Makens, 1964; Bello Acebrón, and Calvo Dopico, 2000), or even cassette players (Dodds and Monroe, 1985, see Rao and Monroe, 1989 for a review). What's more surprising, however, is that our preferences for more expensive goods seem to hold even in cases where the price is arbitrary. For example, Plassmann et al. (2008) allowed participants to sample the same wine when it was labeled as either inexpensive ($5 or $10) or expensive ($45 or $90). Participants reported greater experienced pleasure for wines that were labeled as more expensive, even though what they actually drank was the same in both cases. These results suggest that merely labeling a good as more expensive seems to affect the subjective utility a person experiences from that good.

One might be tempted to write off such findings as the result of a strange demand characteristic; perhaps participants self-report that they enjoy expensive options more in order to signal that they're the kind of person who prefers expensive things. However, some evidence suggests that pricing effects may run deeper than mere demand characteristics. First, Plassmann et al. (2008) found that price affected participants' preferences at the neural level; they found that activation in the medial orbitofrontal cortex (mOFC)—an area of the brain thought to encode the subjective reward utility of different stimuli (see review in Levy and Glimcher, 2012)—was higher when participants thought the wine was expensive than when they thought the wine was cheap. These results suggest that participants actually *experienced* the wine as tasting better when it was labeled as expensive than when it was labeled as inexpensive. Second, pricing influences how effective people think a good will be. Shiv et al. (2005) allowed participants to pay different prices for energy drinks and observed how well they performed on a set of mental acuity puzzles. People who had paid more for the drink showed greater energy-boosting effects than those who got the drink more cheaply (for a similar finding, see Waber et al., 2008). This result further suggests that pricing effects appear to go beyond mere self-reported differences in preferences; simply changing the price individuals pay for a drink affects not only how well they think it works, but also its actual effectiveness.

Although much work has shown that price affects people's expectations about a good (see also Rao and Monroe, 1989 for a review), less work has explored how such effects emerge in the first place. One possibility is that our expectations concerning price information stem from our experience with how markets operate. As any economics major knows, markets tend to conform to the rules of supply and demand. People prefer products that are particularly good or effective, and thus demand for such effective products should increase. Companies, therefore, will likely end up charging more for products that are especially effective due to the higher demand for such products. This relationship means that better tasting and more effective products are likely to be more expensive. One possibility, then, is that our experience with markets causes us to develop an association between price and value; we come to implicitly assume that expensive products must actually be valuable because otherwise sellers would have to lower their prices. In this way, one could explain the expectations we described above as an extension of our experience with human-like markets. It is also possible that our experience with markets allows us to develop more explicit theories about how markets work—we may come to develop rich beliefs about the connection between price and value based on our own understanding of markets. A second possibility, however, is that our expectations about the connection between price and value have nothing to do with our experience in markets. Instead, our preference for more expensive items may stem from more domain-general processes, ones that are not specific to monetary values or markets.

Are our expectations regarding price and value motivated by a domain-general mechanism or by experience with human-like markets? One way to distinguish between these alternatives is to test a population that does not have rich experience with human markets. Here, we test just such a population—capuchin monkeys (*Cebus apella*).

Although turning to capuchins may seem at first glance a strange way to test the mechanisms underlying pricing effects in our own species, there are several reasons why this population is well-suited for this question. First, researchers have successfully used capuchins as subjects in economics studies examining the origins of judgment and decision-making biases (Chen et al., 2006; Egan et al., 2007, 2010; Addessi et al., 2008; Lakshminarayanan et al., 2008, 2011). In many of these studies (e.g., Chen et al., 2006), monkeys were trained to trade tokens with a human experimenter for different kinds of food. Monkeys were then allowed to enter a market in which they had to choose between different experimenters who sold different goods at different prices. Using this market method, researchers have observed that capuchins appear to have many of the same strategies and biases as humans (see review in Santos and Chen, 2009). Like humans, capuchins exhibit endowment effects (Lakshminarayanan et al., 2008), loss aversion (Chen et al., 2006), reflection effects (Lakshminarayanan et al., 2011), and choice-induced preference reversals (Egan et al., 2007, 2010). Given that capuchins show many of the same economic biases as humans, it makes sense to examine whether this species shows pricing biases as well.

In addition, recent work suggests that capuchins seem to understand some aspects of price in the context of their experimental market. Chen et al. (2006) tested whether monkeys' choices in their market obeyed the tenets of standard price theory (see Becker, 1962). Capuchins were asked to allocate a set of tokens across two different kinds of food (e.g., apples and grapes) at a cost of one token per food item. Chen and colleagues then introduced a compensated price shift, in which the price of one of the goods dropped (e.g., a subject now received two apples per token rather than one). The researchers then tested whether monkeys switched their consumption after this compensated price shift; did monkeys buy more of the cheaper good after the price change? Chen and colleagues observed that subjects attended to price information, buying more of the cheaper good after the price shift. These results suggest that monkeys' choices in this market obey standard price theory, and thus that monkeys attend to price information in their market in some of the same ways as human consumers do in real markets.

Because capuchins appear to understand price information in token markets, this species can provide a particularly useful test case for distinguishing between the two different accounts of pricing biases described above. Although capuchins seem to understand certain aspects of pricing information in a token economy (e.g., Chen et al., 2006), they lack human-like experience with how price works in real markets. The capuchin token economies differ greatly from those of human participants, particularly with regard to the connection between a good's potential value and its price. If human-like market experiences are indeed necessary for the development of an association between price and value, then capuchins should not show the same kinds of pricing effects as humans do.

In the current studies, we developed a series of experiments to determine whether capuchins show human-like price biases. Capuchin subjects were taught the price of two novel foods in the context of their token economy (see Chen et al., 2006). We then assessed monkeys' preferences for the two goods in the absence of tokens (i.e., during free choice). If monkeys exhibit human-like pricing effects, then they should prefer the more expensive good to the cheaper good when they have a chance to *freely choose* without paying with their tokens.

Experiment 1 began by teaching capuchins prices for two new foods: differently colored flavored ices. We then allowed monkeys to freely choose between the two colors and tested whether they spontaneously preferred the good that we had told them was more expensive.

## Experiment 1

### Materials and methods

We tested seven brown capuchin monkeys (AG, AH, FL, HR, MD, MP, NN) from the population at the Comparative Cognition Laboratory at Yale University (New Haven, CT). All monkeys had participated in a variety of experiments involving making decisions in their token economy (Chen et al., 2006; Lakshminarayanan et al., 2008, 2011). All studies were approved by the Yale Institutional Animal Care and Use Committee.

Testing was conducted in a cubical testing chamber (75 × 75 × 75 cm), which monkeys entered via a sliding door attached to their main large social enclosure. Only one monkey was allowed into the testing area at a time. Monkeys were free to walk into an adjacent section of the enclosure during testing where no other monkeys were present. Two panels on opposite sides of the testing enclosure allowed participants to interact with the experimenters. Each of the panels had two trading holes (5 × 9 cm), spaced such that the participants could reach through one but not both of the openings at the same time (approximately 25 cm apart). During testing, subjects were allowed to choose different foods presented on a small table with a sliding component that was hooked to the outside of the testing enclosure. The sliding component had two trays each positioned to line up with a trading hole when slid up to the chamber, allowing the subject easy access to their contents. Monkeys were presented with 12 tokens (1 inch diameter aluminum disks) which they could use to “purchase” small Flavor-Ice ice chunks (2.5 × 1 cm) of different colors (orange and blue).

**Figure 1:**
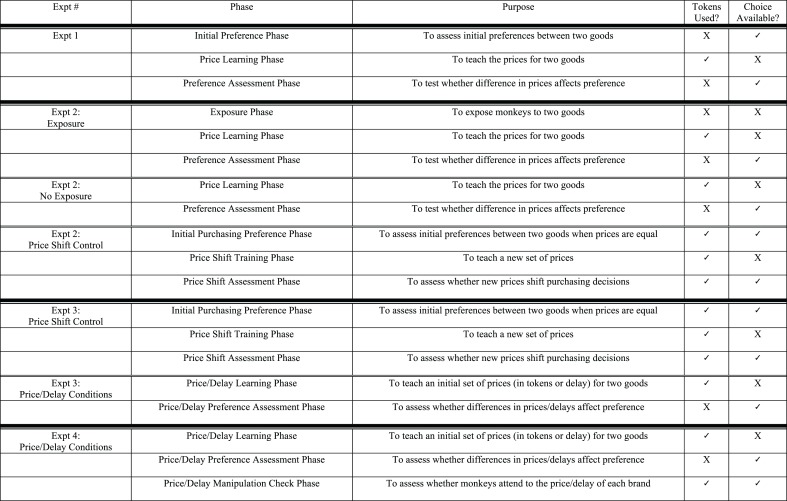
**Description of different experimental conditions and phases**.

Before testing began, each monkey was given one piece of each ice color in order to familiarize them with the taste. The order of color presentation was counterbalanced across monkeys. After familiarization with the different flavors, monkeys began the study. All monkey subjects began on an *initial preference phase*, followed by a *price learning phase*, and then a *preference assessment phase* (see Figure 1 for more details).

In the *initial preference phase*, subjects were given a choice between equal quantities of the blue and orange ice. The goal of this phase was to assess monkeys' initial preference between the two colors of ice. We also wanted to be sure that monkeys did not have strong aversion to one ice color; those monkeys who showed significant preferences were removed from the study. Note that because we did not introduce any tokens during this phase, monkeys had the chance to sample the ices before learning about their prices. During each trial, monkeys were given a choice between the two ices. To do so, the experimenter slid the sliding component away from the testing chamber, placed each piece of ice on its respective tray, and then pushed the sliding component back to allow the subject to choose. The subject then selected one of the two ices; after the subject made its choice, the sliding component was withdrawn and the other ice was removed. We presented subjects with two sessions of 12 trials each, with ice placement counterbalanced across trials. Sessions were run on separate days in order to prevent ice satiation.

After the initial preference phase, subjects moved on to the *price learning phase*. The goal of this phase was to use the token economy to teach monkeys that one of the two goods could be bought at a discount relative to the other. On each trial, monkeys had a chance to give a token to an experimenter who would return either one piece of one color of ice (the *expensive* ice), or three pieces of the other color of ice (the *cheap* ice). Note that the expensive color of ice was priced at three times the price of the cheap color of ice. We chose this difference in price because previous work has shown that monkeys are able to distinguish items that are three times the value of other items (e.g., vanMarle et al., 2006). We conservatively chose which ice was cheaper based on monkeys' initial preferences; monkeys who indicated an initial preference for orange ice (AG, FL, MD) were taught that orange ice was cheap while those that indicated a preference for blue ice (AH, HR, MP, NN) were taught that blue ice was cheap. Testing proceeded as in previous token trading procedures (e.g., Chen et al., 2006; Lakshminarayanan et al., 2008). Specifically, subjects could “purchase” one of the two ices by placing a token into the hand of an experimenter. At the start of each trial, the experimenter placed his hand open to receive the monkey's token. At the same time, he displayed a small dish holding the amount of ice to be traded. Upon receiving a token from the monkey, the experimenter brought the dish up to allow the monkey access. Once the monkey had eaten all the ice, the dish was reloaded, and the next trial began. Each monkey received two sessions of 12 trials with the order of the first color counterbalanced across sessions.

Following the price learning phase, monkeys moved on to the *preference assessment phase*. The goal of this phase was to see if learning the price of the two ices affected the monkeys' preferences for each of the two colors. To test this, we gave the monkeys a free choice between the two colors (i.e., they could take the ices without having to purchase them using their tokens). The preference assessment phase was identical to the initial preference phase; monkeys again received a free choice between the two colors on the sliding trays. Importantly, the experimenter presenting the trays to the monkey was blind to which ice color had been cheap and which had been expensive, and thus could not influence the monkeys' choices. Assuming monkeys were indifferent between the two ice colors initially, we could test whether monkeys show human-like price effects by examining whether they reliably chose the color shown to be more expensive in the price learning phase when choosing in the preference assessment phase.

### Results

We first tested to see if monkeys had an initially strong preference for one of the two colors of ice in the initial preference phase. Two of the monkeys showed a strong and significant initial preference (HG: 12.5%, *p* = 0.0003, JM: 25.0%, *p* = 0.02) and thus were dropped from further testing. All other monkeys did not show a significant preference (FL: 29.2%, *p* < 0.064; AG: 37.5%, *p* = 0.31; AH: 41.7%, *p* = 0.54; HR: 50.0%, *p* = 1.00, MD: 37.5%, *p* = 0.31; MP: 29.2%, *p* < 0.064; NN: 41.7%, *p* = 0.54; all tests exact binomial probability estimates against chance), suggesting that these subjects had no initial preference between the two colors and thus could be used as subjects in the subsequent phases.

Monkeys then went on to learn about the price of the two ices during the price learning phase. Note that in this phase monkeys had no choices—they received equal numbers of trades across the cheap and expensive goods. After learning about the prices, we again tested the monkeys' preferences in the preference assessment phase. Specifically, we compared monkeys' preference for the expensive color in the test condition with their preference for that color before they learned the price information. To do this, we used a repeated measures ANOVA with the color used as expensive as a between subject variable (orange or blue) and time of choice as a within subject variable (before price information and after price information). We observed no main effect of color [*F*_(1, 5)_ = 0.328, *p* = 0.591], suggesting that monkeys had no strong preference for one color over another. We also, however, observed no main effect of timing [*F*_(1, 5)_ = 0.527, *p* = 0.50]. Monkeys' preferences did not change after learning that one item was more expensive. Non-parametric tests confirmed this finding that there was no effect of price training (Wilcoxon signed rank: *Z* = 0.94, *p* = 0.35). We also observed no interaction between color and price [*F*_(1, 5)_ = 0.004, *p* = 0.95].

### Discussion

In Experiment 1, we taught monkeys that one color of ice was three times more expensive than the other, and explored whether monkeys subsequently preferred the more expensive kind of ice when they had free access to both options. In contrast to what's often been observed in humans (Plassmann et al., 2008), monkeys showed no preference for the more expensive ice. Learning which kind of ice was more expensive in the price learning phase did not seem to affect monkeys' preferences in the preference assessment phase. This result suggests that learning that a food is expensive doesn't seem to make monkeys like it more.

There are, however, a few problems with this study. The first concerns whether monkeys noticed the differential pricing of the two goods. Previous work has shown that this population of capuchins understands the “price” of different goods when such goods are sold in different amounts for a single token (Chen et al., 2006). Experiment 1 assumed that similar presentations of different amounts of food would teach monkeys the specific price of each good. It is possible, though, that monkeys did not attend to this information. In Experiment 2, we add a control condition to test that monkeys attended to the pricing information, testing the monkeys on a price shift condition similar to that used in Chen et al. (2006)'s original study.

A second potential flaw in Experiment 1 is that sampling a food may establish a preference that doesn't change once new price information is learned. In Experiment 1, we first exposed monkeys to both flavors of ice in order to obtain a baseline for their preferences. Unfortunately, it is possible that this experience allowed monkeys to establish a preference that could not be changed by price information. In Experiment 2, we directly explore this possibility by varying whether monkeys had previous experience with the foods whose prices were being manipulated. This manipulation allowed us to examine whether prior experience anchored preferences and contributed to the fact that we did not observe a human-like pricing bias in Experiment 1.

## Experiment 2

### Materials and methods

We tested 8 capuchins (AG, AH, FL, HG, HR, JM, MP, NN) from the same colony. All but two monkeys (HG and JM) had previously participated in Experiment 1. One monkey who was tested in Experiment 1 (MD) was not included in this study due to a disinterest in entering the enclosure for testing during the period when this study was run.

We used the same testing enclosure as in Experiment 1, but with a couple of key differences. Instead of using flavored ice as in Experiment 1, Experiment 2 used pieces of differently flavored Jell-O brand gelatin (roughly 1 cm across and 0.65 cm deep). To standardize the shapes of the gelatin, we made each piece using a standard mold. We used six different color/flavor/shape combinations (pink watermelon squares, purple grape crescents, green lime stars, blue blueberry hearts, red strawberry clovers, and yellow lemon triangles). Hereafter each gelatin will be referred to by color. Gelatin colors and experimenter were counterbalanced across subjects.

The procedure of Experiment 2 was similar to that of Experiment 1 except that (1) we explicitly varied the exposure monkeys had to the gelatin and (2) we included a condition to directly test whether monkeys encoded the price information in this study. Each monkey was run on three separate conditions: first, an *exposure condition* and a *non-exposure condition* (presented in a counterbalanced order) followed by a *price shift control condition* (see Figure 1 for more details). In both the exposure and non-exposure conditions, monkeys would ultimately get a choice between two colors of gelatin, one which was shown to be expensive and one which was shown to be cheap.

In the exposure condition, monkeys began with the *exposure phase*, consisting of two sessions. The goal of this phase was to allow the monkeys to systematically taste each of the two gelatin colors. On each trial, monkeys interacted with an experimenter who handed them a single color of gelatin. During each trial, the experimenter began by positioning herself outside either side of the test enclosure, displaying a small dish holding the gelatin to be delivered. The experimenter then brought the dish up to the trading hole, allowing the monkey full access to its contents. Once the monkey had eaten the gelatin, the dish would be reloaded, and the next trial would begin. Each monkey received two sessions of 12 trials (6 for each color gelatin) with the order of the first color gelatin given counterbalanced across sessions.

After subjects completed the exposure phase, they moved on to a *price learning phase*, identical to that of Experiment 1, in which subjects were taught the prices for two colors of gelatin (either blue vs. green or red vs. yellow depending on the counterbalance). In this phase, capuchins participated in a series of five sessions of 12 trials. Each monkey was taught that one color was cheap (i.e., they received three pieces of that color gelatin for a single token), while the other color was expensive (i.e., they received only one piece of gelatin for a token).

After learning the price of the new goods, capuchins then were given a *preference assessment phase*, similar to the one used in Experiment 1, in which subjects were allowed to freely choose between the two colors of gelatin for which they had just learned prices in the price learning phase. Monkeys received 5 sessions of 12 trials. On each trial, monkeys interacted with an experimenter, blind to the nature of the cheap and expensive gelatin goods, who held two small dishes, each containing a colored gelatin piece. The experimenter then simultaneously brought each dish to a separate trading hole, and allowed the monkey to choose one. After the monkey chose the color to consume, the other dish was withdrawn, and the trays were reloaded. Again, the goal of this phase was to determine which of the two flavors the monkeys preferred when they got to freely choose, and to see if price information affected that preference.

In the non-exposure condition, monkeys proceeded through exactly the same phases as in the exposure condition except that we did not include the exposure phase; monkeys only went through a price learning phase and a preference assessment phase. In this way, monkeys in the non-exposure condition had never been exposed to the two novel gelatin colors when they first learned about the gelatins' prices in the price learning phase. If previous exposure to the novel foods had anchored the monkeys' initial preferences in Experiment 1, then wouldn't expect a similar effect here since the goods were totally novel at the point the monkeys learned their prices.

After subjects completed both the exposure condition and the non-exposure condition, they then moved on to the *price shift control condition.* The goal of this condition was to be sure that monkeys learned the different prices. We used a version of the price shift studies used in Chen et al. (2006): monkeys were presented with an *initial purchasing preference phase* (to test the monkeys initial preferences between two colors of gelatin when those colors were the same price), a *price shift training phase* (in which we taught the monkeys a new price for one of the two goods), and a *price shift assessment phase* (to see whether the monkeys responded rationally to this shift in price and allocated their budget accordingly).

The *initial purchasing preference phase* involved assessing the monkeys' initial preferences between the two colors of gelatin being offered when monkeys got equal amounts of the two colors for a single token. Monkeys received five sessions of 12 trials each, with one session run per day. At the beginning of each session, the subject monkey was given 12 tokens with which to buy the gelatin. Experimenters positioned themselves on opposite sides of the testing chamber. One experimenter consistently dispensed pink gelatin, while the other dispensed purple. Both experimenters began the session with their backs facing the monkey. Then, on a synchronized count, both turned around, offering one hand toward a trading hole to receive a token from the monkey, and displaying the gelatin that they offered in the other hand. The monkey was then able to choose the color he preferred by depositing a token in one of the experimenters' hands. The chosen experimenter then presented his tray up to the trading hole so that the monkey could reach the gelatin while the other experimenter would turn his back around in order to indicate that they were no longer available for trade. After the gelatin was completely consumed, experimenters would reload their trays, switch sides and proceed with the next trial. Gelatin colors were counterbalanced across monkeys.

After we had taught monkeys that the two kinds of gelatin were equal in price, subjects moved into the *price shift training phase*. Here the monkeys learned that the prices that they were originally exposed to had changed. Specifically, the good that the subject had liked least (purple: AG, AH, HG, HR; pink: FL, JM, MP, NN) was discounted such that one token went from buying a single piece of gelatin to buying three pieces. As in the initial assessment, monkeys were given 12 tokens to allow them to purchase gelatin from the two experimenters. However, in this case monkeys did not have a choice; on each trial, only one experimenter was available to trade. Both experimenters began each trial with their backs to the testing chamber, but only one experimenter turned at a time, thus providing only one person with whom the monkey could trade. One experimenter offered three pieces of the “discounted” gelatin for a single token, while the other offered the original price of one gelatin piece per token. Each monkey received three sessions of this training before moving onto the price shift assessment phase.

In the *price shift assessment phase*, we tested whether monkeys responded to the change in price they just witnessed by switching their consumption to the cheaper good (see Chen et al., 2006 for another version of this price-shift test). The assessment was similar to the initial purchasing preference phase except that monkeys had a choice of the two gelatin colors now at the new prices. If monkeys accurately attend to and track price, they should switch their consumption to the less costly good in this assessment phase. Each monkey completed 5 sessions of 12 trials.

### Results

We first analyzed how monkeys performed on the price shift assessment as compared to the initial purchasing preference phase. Did subjects successfully understand the more expensive price and therefore choose the cheaper good when they had to pay for it? To test this, we ran a repeated measures ANOVA with price (initial purchasing preference phase with equally priced goods vs. price shift assessment where one good was more expensive) as a within subjects factor and color of good chosen to be cheap (pink or purple) as a between subjects factor. We observed no effect of color [*F*_(1, 6)_ = 0.25, *p* = 0.879]. We did however observe a significant main effect of price [*F*_(1, 6)_ = 123.85, *p* < 0.0001]. Although monkeys overall didn't have a preference for either color initially [*t*_(7)_ = 0.944, *p* = 0.377], monkeys preferred the cheaper good after the price change [81.0% preference for the cheap good: *t*_(7)_ = 5.89, *p* < 0.0006]. All monkeys spent more on the cheap color after the price shift than before it (Paired sign test: *p* = 0.0078). We also observed a small interaction of color and price [*F*_(1, 6)_ = 7.67, *p* = 0.03]; monkeys showed more of a shift toward the cheaper good when pink was the cheap good than when purple was the cheap good. Overall, these results demonstrate that subjects recognized the price at which each color gelatin was being offered and attended to this information in their choices. Importantly, all monkeys consumed more of the cheaper good after the price shift as one might expect given standard price theory (see Chen et al., 2006).

We then tested whether monkeys chose the expensive good above chance in the preference assessment phases of the exposure and non-exposure conditions. As in Experiment 1, we observed no effect of price in Experiment 2; monkeys chose the expensive good on average 53.1% of trials [One-sample *t*-test: *t*_(7)_ = 0.431, *p* = 0.68]. This chance-level performance was true for both the blue/green color combination [48.5% choice to expensive, *t*_(7)_ = 0.26, *p* = 0.80] and the red/yellow color combination [57.7% choice to expensive, *t*_(7)_ = 0.718, *p* = 0.50]. Despite the fact that monkeys robustly understood price in the price shift control, that information didn't seem to affect their preferences or how much they valued each kind of gelatin when they got to freely choose one.

We also looked to see whether initial experience affected the magnitude of price effects using a repeated measures ANOVA with exposure level (exposure condition vs. non-exposure condition) as a within subject factor and the color combination subjects had experience with (blue/green vs. red/yellow) as a between subjects factor. We observed no effect of experience [*F*_(1, 6)_ = 1.198, *p* = 0.316]; subjects showed just as strong a preference for the expensive good in the exposure condition (Mean = 57.9% preference for the expensive good) as they did in the non-exposure condition (48.3%).

### Discussion

We had two goals in Experiment 2. Our first was to confirm that monkeys attended to the prices that we had presented. To do this, we performed a price shift like that of Chen et al. (2006), changing the price of one color of gelatin to a “sale price” that was three times cheaper than the price of the other gelatin. Our subjects overwhelmingly purchased more of the cheaper gelatin, as they had done in previously published studies (Chen et al., 2006). This result indicates that capuchin monkeys do attend to the price of the goods presented in this token exchange market. In addition, this result also demonstrates that monkeys use price as a factor in their purchasing decisions in this experimental market.

The second goal of Experiment 2 was to examine the role of prior experience in the monkey's preferences—specifically, to find out whether prior experience may have moderated any effect that price may have on preferences. Regardless of exposure, capuchins did not prefer the more expensive good; as in Experiment 1, no monkey showed a preference for either the cheap or expensive good. This new result indicates that monkeys' lack of a preference for the more expensive good in Experiment 1 is not because of anchoring due to prior exposure. Instead, the results of Experiment 2 suggest that regardless of whether monkeys have previous experience with a particular food, capuchins base their preferences on their subjective experience with a food, rather than any external price information.

A possible issue with Experiments 1 and 2 is that both studies used foods that were different colors and—perhaps more importantly—different flavors. It is possible that price information did not influence monkeys preferences in these studies because monkeys may have had slight (although not statistically significant) preferences based on the colors and flavors of the foods we offered them. These initial preferences may have overshadowed any changes in valuation that occurred due to differences in price. The results of Experiments 1 and 2 therefore leave open the possibility that monkeys may prefer the more expensive of two *perfectly equal* goods. To examine this, Experiment 3 tested monkeys' preferences for differently priced yet perceptually identical foods, just as has been done in human pricing effect experiments (e.g., Plassmann et al., 2008). To do so, we used two of the same kind of food (pieces of Kix cereal) to ensure that the two goods were perfectly equal. However, to be sure that the individual foods were distinguishable in some way, we paired the pieces of cereal with novel “brand” logos. In this way, Experiment 3 was able to allow monkeys to choose between foods which would be identical in perceptual experience (i.e., taste) yet could have different prices.

Experiment 3 also aimed to explore whether *any* other factors could affect monkeys' preferences for different foods. Given that monkeys failed to show pricing effects in Experiments 1 and 2, we hoped to find another factor that could affect monkeys' preferences even if this species lacks pricing effects. One external factor that is unrelated to price but appears to play a role in humans' reward preferences is the wait time that comes with different rewards. Although we tend to dislike waiting for a reward (e.g., Berns et al., 2007) and find long wait times very costly, we also find rewards more enjoyable if we have to wait longer for them (e.g., Alessandri et al., 2008a). This so-called “delay justification effect” has also been observed in 7 year-old children, who also prefer a stimulus that usually follows a delay to a stimulus that usually does not follow a delay (Alessandri et al., 2008b). Although there is some controversy about the mechanisms underlying these delay justification effects (see Festinger, 1957 vs. Zentall, 2010), it seems clear that our tendency to overvalue stimuli that are associated with longer delays may be part of a larger, more general tendency to prefer stimuli associated with more cost or effort, regardless of whether such effort comes in the form of extra waiting (e.g., Alessandri et al., 2008a), more difficult work (e.g., Festinger and Carlsmith, 1959), or even more embarrassment (e.g., Aronson and Mills, 1959).

Interestingly, humans are not the only species to experience delay and other forms of effort justification effects. Recent comparative work suggests that some non-human species also prefer a stimulus associated with additional cost or effort (Clement et al., 2000; Kacelnik and Marsh, 2002; Friedrich and Zentall, 2004; Gipson et al., 2009), particularly in cases when delays are involved (DiGian et al., 2004; Pompilio and Kacelnik, 2005; Zentall and Singer, 2007; Wanat et al., 2010; see Zentall, 2010 for a review). For example, DiGian et al. (2004) presented pigeons with two stimuli that predicted an immediate reward: one stimulus was available immediately and one stimulus appeared only after a 6 s delay. When given a choice between these two reinforcing stimuli, pigeons reliably preferred the stimulus that appeared after a delay even though it predicted the same kind of reward as the stimulus that was available immediately. This result suggests that pigeons find a stimulus more rewarding if they have to wait longer for it. In this way, other species appear to value goods more highly the longer they have to wait for them^1^. We therefore wanted to see whether capuchins might use delay as an extrinsic factor that mediated their preferences for different kinds of foods, even though they don't incorporate price information into their preferences.

Experiment 3 tested whether varying either a food's wait-time or its price would affect monkeys' preferences when freely choosing between different options. The similarity between these two dimensions—delay and price—allowed us to set up two methodologically identical studies testing each of these factors. We taught monkeys about two novel brands' prices (one piece for one token or three pieces for one token) or wait times (available after 30 s or immediately available) through repeated exposure, and then allowed the monkeys to choose between the two brands. If longer delays affected capuchins' preferences more so than higher prices, then monkeys should prefer brands that come after a long delay even though they show no preferences across differently priced brands. This type of effect would imply that capuchins' preferences in a token economy can in fact be influenced by extrinsic properties, but that price information is not one of these properties. On the other hand, if the capuchins show neither delay nor price effects, this finding would suggest that capuchins may evaluate goods based entirely on their subjective experience with them, rather than any additional extrinsic features.

## Experiment 3

### Materials and methods

Subjects were 7 adult brown capuchin monkeys (AH, FL, HG, HR, JM, MD, NN) from the same colony. Two monkeys who had previously participated in both Experiments 1 and 2 were excluded from Experiment 3: one low-ranking monkey (AG) was not included due to social problems in the colony during the time of testing, while another monkey (MP) was not included due to a disinterest in entering the enclosure for testing during the period when this experiment was run.

Experiment 3 used pieces of Kix cereal (General Mills, US)—a familiar food for these subjects—as a reward. To differentiate between the cheap and expensive versions of the cereal, we created three pairs of “brands” which were denoted by three easily distinguishable pairs of symbols: red flower vs. yellow star, green clover vs. yellow moon, and orange balloon vs. blue horseshoe. These brand symbols (approximately 13 × 13 cm) were displayed prominently on the white shirt of the experimenter that consistently offered that brand. The brand symbol was also displayed on the container (a 3 oz clear plastic drinking cup with the front half cut out to make food easily reachable) from which the cereal was dispensed. Each experimenter presented the cups to the monkey on white foamcore platforms that were covered in white duct tape for ease of cleaning. Each cup always contained a single piece of cereal, but each platform could hold one to three cups depending on condition. We attached the cups to the platform using Velcro to keep them stable. Each monkey always saw the same experimenter associated with each brand, but the brand and experimenter were counterbalanced across monkeys.

Each monkey participated in three conditions: a *price shift control condition*, *a delay condition* and *a price condition* (see Figure 1 for more details). The price shift control condition was administered first, with the delay and price conditions presented afterwards in a counterbalanced order.

The *price shift control condition*, which was nearly identical to the price shift control condition used in Experiment 2, was used to ensure that monkeys could attend to price information in the context of the branded cups used in Experiment 3. If monkeys are able to accurately track the prices of brands, then—as in Experiment 2—they should buy more of the cheaper brand after the price shift. After all of the monkeys had completed the price shift control, demonstrating that they were paying attention to the price of the cups and using this price information to make decisions, they then moved on to either the price condition or the delay condition (in a counterbalanced order).

As in the non-exposure condition of Experiment 2, the *price condition* consisted of two phases: a *price learning phase* (where monkeys were exposed to the prices of two new brands), and *price preference assessment phase* (where monkeys were allowed to choose between the two brands in the absence of price). The goal of the *price learning phase* was to expose the monkeys to the prices of two new brands of cups. One of the two brands was cheap (i.e., one token could purchase three cups with one piece of Kix each), while the other was expensive (i.e., one token could purchase only one cup with one piece of Kix). Note that this is identical to the procedure used for different goods in Experiments 1 and 2, except that we used different brands instead of different foods. Monkeys each received four sessions, each consisting of 16 trials each. In each trial, the two branded experimenters dropped a token into the enclosure in unison, before moving to opposite sides of the enclosure. Then one of the two experimenters turned around, offering to trade the contents of her cup(s) in exchange for a token. After the monkey paid one of the two experimenters and received the food, the two experimenters switched sides and repeated the process.

Monkeys then moved on to the *price preference assessment phase*. The goal of this phase was to determine whether capuchins changed their preference for the two brands based on the price information they had just been taught. Each price preference session began with four price reminder trials identical to the original price learning trials. We added these reminder trials to be sure that monkeys accurately remembered which brand was which. After these reminder trials, a different experimenter (who was blind to condition) gave the monkeys a choice between the two different branded cups in the absence of any tokens. Importantly, in this case, each branded cup only contained a single piece of Kix. If monkeys had developed a preference for a specific brand based on the pricing information, then they should selectively choose that preferred brand when given a free choice to eat food from either brand. Monkeys received four sessions of 16 trials each. If capuchins use price as an indicator of quality, then they should selectively prefer the previously expensive brand (the one for which they had previously been offered one cup for a single token) to the previously cheap brand (the one for which they previously received three cups for a single token).

The *delay condition* mirrored the price condition with one key difference: instead of varying the price of the two brands, we instead varied the time the monkeys needed to wait in order to receive each brand. Like the price condition, the delay condition involved two phases: a *delay learning phase* and a *delay preference assessment phase.* The *delay learning phase* served to teach the monkeys that one brand was associated with a delay and one was not. Specifically, one of the two brands (the “expensive delay” brand) was associated with a 30 s delay while the other brand (the “cheap delay” brand) was given immediately. Each monkey received four 16-trial sessions. On each trial, the two branded experimenters dropped a token into the enclosure in unison before moving to opposite sides. Then, one of the two experimenters turned around, offering to trade with the monkey. When the monkey gave a token to the experimenter holding the cheap brand, she moved her cup toward the trading hole immediately, allowing the monkey to immediately retrieve the piece of Kix in the cup. In contrast, when the monkey gave a token to the experimenter holding the expensive brand, she waited 30 s before moving her cup toward the trading hole, thus requiring the monkey to wait before retrieving the piece of Kix in the cup.

Following the *delay learning phase*, monkeys moved on to the *delay preference assessment phase*. The goal of delay preference assessment phase was to determine whether the relative cost of the two brands in terms of time had affected the monkeys' preferences between the two. The structure of these sessions was identical to the sessions presented in the price preference assessment phase except that we varied the brands' delay times rather than prices. During each trial, an experimenter who was blind to which brand had previously been associated with the delay turned to face the enclosure, offering a cup from one of the brands at each of the two trading holes. If capuchins come to value brands that are associated with a greater wait time, then they should selectively prefer the brand that was previously associated with the delay over the brand that was previously available immediately. On the other hand, if capuchins do not use increased delay as an indicator of quality, then they should show no preference in the delay preference assessment phase.

### Results

We first explored whether monkeys had an initial preference for one of the two brands in the price condition when they initially encountered them in purchasing preference phase. None of the seven monkeys showed a statistically significant preference across the two brands (percent choice to the brand that would later be made cheap: HR: 48%, *p* = 0.90; FL: 48%, *p* = 0.90; NN: 42%, *p* = 0.26; MD: 38%, *p* = 0.059, JM: 41%, *p* = 0.17; HG: 47%, *p* = 0.71, AH: 50%, *p* = 1.00).

After the price shift, however, all the monkeys developed a significant preference for the cheaper of the two brands: (HR: 98%, *p* < 0.0001, FL: 97%, *p* < 0.0001; NN: 94%, <0.0001; MD: 98%, <0.0001; JM: 95%, <0.0001; HG: 95%, <0.0001; AH: 70%, *p* = 0.0016). A paired *t*-test revealed that monkeys as a group preferred the cheaper brand when using their currency [*t*_(6)_ = 9.83, *p* < 0.0001]. Again, this result suggests that monkeys do use price information when distinguishing between brands, actively shifting their consumption to the cheaper brand when they need to pay tokens to obtain it.

Given that all monkeys correctly paid attention to the price of these brands of cups, we then explored whether teaching monkeys that the brands had different prices had an effect on their preferences for each brand. Unfortunately, three subjects did not fully complete testing due to social problems in the enclosure. We therefore ran our analysis on just the four monkeys that completed all the tests. As in previous experiments, we presented monkeys with two new brands and tested whether monkeys chose the more expensive one above chance in the price preference assessment phase. We again saw no effect of price on monkeys' preferences. As in all previous experiments, no monkeys showed a significant preference for the expensive brand over the cheap brand (percent choice to expensive brand: HR: 50%, *p* = 1.0, FL: 48%, *p* = 0.90; NN: 48%, *p* = 0.90; MD: 53%, *p* = 0.71).

We then tested whether monkeys showed a preference for delay—whether they chose the expensive delayed brand above chance in the delay preference assessment phase. Interestingly, we also saw no significant preferences for the brand with the expensive delay. One monkey, HR, did show a significant preference, but her pattern of performance went in the opposite direction than we hypothesized—HR significantly preferred the immediately available cheap brand (20%, *p* < 0.0001). All other monkeys chose between the immediate and delayed brand at chance (FL: 47%, *p* = 0.71; NN: 48, *p* = 0.90; MD: 55%, *p* = 0.53).

### Discussion

Experiment 3 had two main goals. The first goal was to examine whether using two goods that are experientially identical but perceptually distinguishable would cause monkeys to show a price preference that they do not otherwise show. In contrast to Experiments 1 and 2, which used different flavors of the same food, Experiment 3 used identical cereal pieces as the reward for both the expensive and cheap options. In spite of this change, monkeys still showed no preference between the two brands during price preference assessment trials. Importantly, all monkeys successfully switched to the cheaper good when it went on “sale,” again indicating that they were attending to the price information we had shown them. This finding implies that the monkeys' lack of preference in the first two experiments is likely not due to differences in the two foods being offered, as monkeys' failure to use price as an indicator of value persists even when identical items are offered as goods.

The second goal of Experiment 3 was to explore whether *any* factors could affect monkeys' preferences for different foods. To this end, we explored whether the amount of time monkeys were required to wait for one brand over the other affected their preferences. Rather than teaching monkeys different prices, the delay condition of Experiment 3 taught the monkeys that they had to pay different costs in terms of time. We found that monkeys did not show a preference for the good previously associated with a delay. This finding shows that the use of delay as a cue to quality may not be as robust as some previous studies had suggested (DiGian et al., 2004; Pompilio and Kacelnik, 2005; Zentall and Singer, 2007; Wanat et al., 2010; see Zentall, 2010 for a review). In this context, it seems that capuchins use neither price nor delay as cues to quality.

Our use of Kix cereal in Experiment 3 had one important drawback. This cereal was already quite familiar to our subjects, as it had previously been used in a number of experiments in the lab. Although Experiment 2 established that prior exposure to a food does not affect monkeys' preferences in terms of price effects, we still worried that it might be difficult for monkeys to think of this food differently based on its brand since it was so familiar to them previously. To solve this issue, Experiment 4 used the same approach as Experiment 3, but with a novel kind of food: Crunch Berries cereal (Quaker Oats, US). The Crunch Berries offer a couple of major advantages over Kix. First, this cereal is available in four colors (red, green, blue, and purple) giving us the ability to make the two different brands perceptually different and thus easier to discriminate. However, all four of colors of Crunch Berries taste the same; in this way, there is no reason that subjects should form a strong preference for one color over another. Second, our subject monkeys had no outside experience with Crunch Berries, and thus we were able to ensure that the monkeys' only knowledge of differences between the colors and brands was acquired during the exposure they received during training and testing.

In addition to using a different food reward, Experiment 4 also aimed to be sure that monkeys were able to distinguish between the two brands and make choices based on them. We therefore added more reminder trials at the beginning of each testing session to be sure that the monkeys remembered which brand was cheap vs. expensive and which brand required a delay. To determine whether the monkeys were accurately tracking brands in both the price and delay conditions, we also added a set of manipulation checks to ensure the monkeys were accurately tracking which brand was associated with a higher price/delay.

## Experiment 4

### Materials and methods

Subjects were 6 adult brown capuchin monkeys (FL, HG, HR, JM, MD, NN). Five of the monkeys tested (FL, HG, HR, JM, NN) had previously participated in Experiment 3. Two monkeys (HG, MD) who participated in Experiment 3 were excluded from Experiment 4 due a disinterest in entering the enclosure for testing during the period when this study was run. One monkey (MP) participated in Experiments 1 and 2, but not in Experiment 3.

We used four Crunch Berry cereal colors (red, blue, green, purple) as goods; these colors all tasted the same so monkey should not have had any preferences for colors based on the flavor. We again associated each reward color with an individual “brand” using symbols: red Crunch Berries with a red flag, blue Crunch Berries with a blue sun, purple Crunch Berries with a purple teardrop, and green Crunch Berries with a green snowflake. The brand used in each condition and the experimenter associated with each brand were counterbalanced across monkeys. As in Experiment 3, brands were displayed both on the experimenter's shirt, and the cups containing the Crunch Berries.

In Experiment 4, each monkey participated in 2 conditions: a *price condition* and a *delay condition* (see Figure 1 for more details). As in previous experiments, the *price condition* began with two phases: a *price learning phase* (where monkeys were exposed to the prices associated with two brands), and a *price preference assessment phase* (where monkeys were allowed to choose between the two brands in the absence of price). However, at the end of the price condition, we added an additional phase, a *price manipulation check phase*.

In the *price learning phase*, monkeys were taught that one brand was expensive (for each token the monkeys got only one cup/Crunch Berry), while the other brand was cheap, (for each token the monkeys got three cups/Crunch Berries). The price learning phase consisted of one session with 16 trials with procedures identical to the price learning phase of Experiment 3.

After monkeys completed the price learning phase, they moved on to the *price preference assessment phase*, where we aimed to determine whether the prices of the two brands impacted preferences. The price preference assessment was performed over four sessions, each consisting of a set of 8 reminder trials (identical to the original price learning phase trials), and 16 test trials. Note that we doubled the number of reminder trials from Experiment 3 to ensure that the monkeys would remember the prices of the two brands. After reminder trials were complete, a different experimenter (blind to condition) gave the monkeys a choice between the two brands of cups in the absence of any tokens. As in Experiment 3, monkeys chose between the two brands of cups now offering one Crunch Berry each. Again, if monkeys developed a preference for the more expensive Crunch Berry brand after learning its price, then they should selectively choose the expensive brand over the cheap brand in these trials.

We then moved on to the *price manipulation check phase.* This phase served to reaffirm that the monkeys were paying attention to the price of the two Crunch Berry brands. Specifically, we expected that when the monkeys had to pay for the two brands using their tokens, they would take price information into account and therefore choose the cheap brand (which gave them the most food). The price manipulation check phase consisted of four sessions, each containing 8 reminder trials (identical to the original price learning phase trials) and 16 manipulation check test trials. In these test trials, the monkeys had to use their tokens and choose between the expensive brand (which gave only one cup/Crunch Berry per token) and the cheap brand (which offered three cups/Crunch Berries per token). These trials began when the two branded experimenters dropped a token into the enclosure before moving to opposite sides. After a synchronized count, the two experimenters turned around, allowing the monkey to choose to trade the token for either the cheap brand or the expensive brand. Monkeys who attend to price should choose to buy the cheap brand more often than the expensive brand since the cheap brand gives them more food overall.

As in Experiment 3, the *delay condition* mirrored the price condition (a *delay learning phase*, a *delay preference assessment phase*, and a *delay manipulation check phase)* with only one key difference: instead of varying the price of the two brands, we instead varied the delay time the monkeys needed to wait in order to receive each brand: one brand was associated with a 30 s delay (the “expensive delay”), while the other was given immediately (the “cheap delay”).

### Results

We first tested whether monkeys showed a preference in the price preference assessment phase. As in all previous experiments, we saw that monkeys as a group did not show a preference for the expensive good [*t*_(5)_ = 1.32, *p* = 0.25]. Two monkeys showed preferences for the color associated with a cheap price (percent choice of expensive: HR: 31%, *p* = 0.004; NN: 36%, *p* = 0.03) and all other monkeys showed no preference (FL: 48%, *p* = 0.90; HG: 42%, *p* = 0.26; JM: 61%, *p* = 0.10; MP: 48%; *p* = 0.90). In the price manipulation check phase, monkeys as a group showed a preference for the cheap good [*t*_(5)_ = 18.81, *p* < 0.0001]. Indeed, all monkeys individually showed a preference for the cheap good (Preference for expensive symbol: FL: 6%, *p* < 0.0001; HR: 0%, *p* < 0.0001; HG: 6%, *p* < 0.0001; JM: 0%, *p* < 0.0001; NN: 0%, *p* < 0.0001; MP: 15%, *p* < 0.0001). Comparing monkeys' performance across the two phases also revealed a significant effect of phase [*t*_(5)_ = 8.86, *p* = 0.0003], suggesting that although monkeys attend to which good is more expensive when they must spend their tokens, the same subjects do not prefer the expensive brand when they can freely choose between the two brands.

We then explored how monkeys performed on the delay preference assessment. Overall, monkeys showed no preference for the delayed good [*t*_(5)_ = 0.74, *p* = 0.49]. One monkey showed a significant preference in the opposite direction (preference to delayed good: NN: 36%, *p* = 0.03), but all other monkeys did not show any statistically significant preference (FL: 61%, *p* = 0.10; HR: 53%, *p* = 0.71; HG: 50%, *p* = 1.0; JM: 44%, *p* = 0.38; MP: 39%; *p* = 0.10). Even though monkeys did not show a preference for the delayed good, as a group they showed a significant preference for the immediate reward during the delay manipulation check phase [*t*_(5)_ = 5.12, *p* = 0.004]. Individually, four monkeys showed a significant preference for the immediate reward (preference to the delayed reward: FL: 4%, *p* < 0.0001; HR: 19%, *p* < 0.0001; JM: 23%, *p* = 0.0002; MP: 8%, *p* = 0.0001), but two other monkeys' performance was not statistically significant (HG: 38%, *p* = 0.11; NN: 35%, *p* = 0.06). Comparing monkeys' performance across the delay preference assessment phase and the delay manipulation check phase revealed a significant effect of phase [*t*_(5)_ = 3.27, *p* = 0.02]. Monkeys attended to which brand was the delayed brand in the manipulation check, yet they still formed no preference for the brand previously associated with delay when they had the chance to get both brands immediately.

### Discussion

We had two main goals in Experiment 4. Our first goal was to examine whether the use of a familiar reward in Experiment 3 had prevented monkeys from show pricing and delay effects. Experiment 4 dealt with this issue by testing whether monkeys showed delay and pricing effects for food rewards that were both unfamiliar and easily distinguished (differently colored Crunch Berries). Despite this methodological change, we observed the same pattern of performance as in Experiment 3: capuchins showed no preference for the more expensive brand in either the price or delay condition.

The second goal of Experiment 4 was to determine whether the monkeys were in fact tracking the prices and delays that were associated with the two different brands. To assess this, we added a manipulation check in both the price and delay conditions. These manipulation checks revealed that monkeys were accurately tracking which brand was expensive in terms of both delay and price, yet the same subjects' preferences were unaffected by these cues.

## General discussion

Across four experiments, capuchins did not moderate their preferences with regard to price. In Experiment 1, capuchins showed no preference for the more expensive piece of ice. In Experiment 2, capuchins showed no preference for the more expensive color of gelatin, regardless of prior exposure. In Experiments 3 and 4, capuchins showed no preference for the more expensive brand of cereal. Across several studies, however, capuchins consistently passed manipulations checks showing that they understood the price associated with each of the two goods involved. Taken together, these findings imply that capuchins do not show a human-like pricing effect—learning the price of a good does not change the capuchins' preference for that good.

In addition, these results suggest that capuchins' preferences may not be affected by other factors—such as delay information—either. Across Experiments 3 and 4, capuchins also failed to update their preferences for different foods based on the delay they were required to wait for that food. This lack of a preference for delayed rewards conflicts with previous studies demonstrating that human and non-human species do—at least in some cases—prefer stimuli associated with additional time or effort (DiGian et al., 2004; Pompilio and Kacelnik, 2005; Zentall and Singer, 2007; Wanat et al., 2010; see Zentall, 2010 for a review). It's worth noting, however, that the general preference for longer delays is likely to be less robust than the preference for higher priced goods—a growing body of studies demonstrate that human and non-human participants do not always show effort and delay justification effects (Vasconcelos et al., 2007; Arantes and Grace, 2008; Shibasaki and Kawai, 2008, 2011; Vasconcelos and Urcuioli, 2009). In addition, there are many situations in which humans and animals tend *not* to show delay justification effects; indeed, the literature on discounting effects in humans and animals suggests that both of these populations often prefer cases in which delays are *shorter* rather than longer (see review of this work in Stevens, 2010). For this reason, it is possible that our subjects might show other effects on value manipulation in future studies despite not showing the delay effects we hypothesized here. Nonetheless, the results of the current study still provide hints that delay and other effects on preferences may be less robust than previously thought.

Another potential problem with our study concerns how monkeys were presented with price information in their experimental token economy. As in previous studies (e.g., Chen et al., 2006; Lakshminarayanan et al., 2011), we communicated the price of a good to monkeys by changing the amount of that good that monkeys received for a single token. While this way of indicating price information has been validated in previous work (see Chen et al., 2006 for evidence that monkeys obey the tenets of standard price theory when tested using this method), it also resulted in a methodological worry: when learning the price of different goods, monkeys always received more of the “cheaper” good than of the more expensive one. In this way, monkeys inadvertently wound up having more experience with cheap vs. expensive items. We attempted to deal with this potential confound in several ways. First, we ran all preference assessment phases on different days than we taught monkeys the price of the different goods. In this way, we hoped that if monkeys became satiated on the cheaper good during the price learning phases, this would not extend to their preference choices since preference assessments were run on separate days than exposure to cheap and expensive goods. In addition, some of our studies used foods that are very familiar to monkeys (e.g., Kix) so that differential exposure in our studies would be trumped by the monkeys' previous experience with these foods. Nevertheless, it's worth noting that this differential exposure across cheap and expensive conditions is one confound in our studies that could potentially have influenced the lack of pricing effects we observed.

Based on the control and experimental conditions used, we can rule out a number of reasons for why the monkeys might not be showing a human-like pricing effect. First, our control conditions demonstrate that monkeys' lack of preference is not due to an inability to understand price. In Experiment 2, subjects showed that they understood the price of the two goods; in the price shift control condition, subjects spent more of their tokens on a good that was “on sale” than on an equivalent good that was not. This replicates previously published findings (e.g., Chen et al., 2006) showing that capuchins can both track price and use price information in their purchasing decisions. In addition, other control conditions revealed that capuchins successfully track the price and delay associated with different brands of goods. In Experiment 4, capuchins chose to buy a cheap brand over an expensive brand, and an immediate brand over a delayed brand, suggesting that subjects successfully use both price and delay information in their purchasing decisions even though they do not use these factors to form their preferences. Finally, our control conditions rule out the possibility that capuchins failed to show pricing effects because their prior experiences anchored their subsequent preferences. In Experiment 2, we established that capuchin monkeys failed to show a price effect regardless of whether they had previous experience with the foods serving as goods.

Overall, then, our findings suggest that capuchin monkeys perform very differently than humans when interacting with differently priced goods. Although humans regularly prefer goods that are higher in price, capuchin monkeys appear to show no such effect. This pattern of performance is relatively surprisingly for two reasons. First, our results suggest that capuchins fail to fall prey to arbitrary price information when deciding between different goods. Our failure to observe pricing effects in capuchins is also surprising in light of the fact that this species exhibits a number of other classic judgment and decision-making biases, such as the endowment effect (Lakshminarayanan et al., 2008), loss aversion (Chen et al., 2006), and the reflection effect (Lakshminarayanan et al., 2011). Indeed, to our knowledge, the price effect is the first judgment and decision-making heuristic to have been studied in non-human primates and not observed. Our results therefore suggest that pricing effects may rely on mechanisms that are distinct from those involved in these other biases. Indeed, our results suggest that pricing effects may be due to cognitive mechanisms or specific experiences that are uniquely human.

One uniquely human experience that could give rise to human-specific pricing effects is our species' practice of participating in markets in which there is often an association between price and value. In a free market, companies can only charge what people are willing to pay for their goods. As such, in most human markets, there will often be an association between a good's price and its actual quality. Humans may thus generalize these experiences to falsely believe the price of an item is *always* indicative of its quality. Under this potential explanation, we might not expect capuchin monkeys to show a similar effect since the markets they trained in are not markets that have associations between price and quality. In this way, our findings have narrowed down the kinds of human-specific experiences that likely lead to price effects.

The goal of these studies was to gain insight into the mechanisms underlying pricing effects in humans. Although we know much about how and when these effects occur, little work to date had addressed where these effects come from in the first place. By comparing our own biases to those of capuchin monkeys, we hope to have shed light on the mechanisms underlying human pricing effects. Indeed, we have observed that—in contrast to other decision-making biases—pricing effects may be uniquely human. Our results therefore hint that monkeys may choose between goods simply based on their experience with different items rather than using the sorts of arbitrary factors that humans use.

### Conflict of interest statement

The authors declare that the research was conducted in the absence of any commercial or financial relationships that could be construed as a potential conflict of interest.

## Footnotes

^1^Nonetheless, there does exist a number of studies suggesting that human and non-human animals do not always show delay and other effort justification effects (Vasconcelos et al., 2007; Arantes and Grace, 2008; Shibasaki and Kawai, 2008, 2011; Vasconcelos and Urcuioli, 2009). For example, in one experiment, Shibasaki and Kawai (2008) observed that people prefer stimuli that follow low-effort tasks than those that follow high-effort tasks. These authors found a similar pattern in Japanese macaques (Shibasaki and Kawai, 2011) in a task in which different stimuli appeared after different numbers of touchscreen presses. Like humans, macaques appeared to show a reverse effort justification effect, statistically preferring the stimulus associated with the least effort over the one associated with the most.
